# Characteristics of working hours and the risk of occupational injuries among hospital employees: a case-crossover study

**DOI:** 10.5271/sjweh.3905

**Published:** 2020-10-30

**Authors:** Mikko Härmä, Aki Koskinen, Mikael Sallinen, Tomohide Kubo, Annina Ropponen, David A Lombardi

**Affiliations:** 1 Finnish Institute of Occupational Health, Helsinki, Finland; 2 National Institute of Occupational Safety and Health, Kawasaki, Japan; 3 Harvard T.H. Chan School of Public Health, Boston, USA

**Keywords:** fatigue, night work, registry data, shift work, shift worker, working time, work hour

## Abstract

**Objectives:**

We investigated the association of working hours with occupational injuries in hospital shift work.

**Methods:**

Registry data of occupational injuries of hospital employees from 11 towns and 6 hospital districts were linked to daily payroll data to obtain working hours for 37 days preceding the first incidence of the injury (N=18 700). A case-crossover design and associated matched-pair interval analysis were used to compare working hour characteristics for three separate hazard windows among the same subjects. Conditional logistic regression was used to calculate odds ratios (OR) with 95% confidence intervals (CI).

**Results:**

We found an elevated risk of an occupational injury for workdays with evening shifts (OR 1.09, 95% CI 1.03–1.14) and workdays following night shifts (OR 1.33, 95% CI 1.17–1.52). After excluding commuting injuries, the risk increased during the evening shifts (OR 1.15, 95% CI 1.09–1.23) and the work days following night shifts (OR 1.44, 95% CI 1.24–1.69), but was no more significant during the morning shifts. Injury risk increased following a week of ≥5 morning shifts or ≥3 evening shifts, but did not increase according to the number of preceding night shifts or quick returns. The length of the work shift (OR 1.22, CI 1.06–1.42) – not the length of the weekly working hours – was associated with an increased risk.

**Conclusions:**

The results indicate an increased occupational injury risk during the evening shifts and during work days following night shifts, with the risk increasing according to the number of evening but not night shifts.

Approximately 20% of European employees and, in some industries, up to 38% of US employees work in shifts (1). Based on the EU Working Time Directive (2003/88/EC), shift work means any method of organizing work in shifts where workers succeed each other according to a certain pattern, entailing the need for workers to work at different times of the day. Shift rotation can be regular or irregular, and the shift rotation can be continuous or discontinuous in relation to weekends. In Europe, irregularly rotating working hours are frequent in health and social care sector, as well as in commerce, hotels, restaurants and catering, all sectors in which female workers dominate (1).

It has been estimated that the costs due to work-related injuries amount to US$ 250 billion a year, which is approximately 1.8% of GDP in the US and 2.7–6.0% of GDP in countries like Norway, Sweden, Finland and Denmark (2). In Finland, there are over 125 000 occupational and commuting injuries annually, based on the obtained claims received by the Finnish Workers’ Compensation Center.

Compared to day work, night and shift work has been reported to be associated with an increased number of occupational injuries in the general population (3), and in industry, construction, mining (4, 5), and health and social care sectors (6–9). Changing the work schedule from day to nonstandard shift work may increase injury risk at work (10). Based on a recent review and meta-analysis of Fischer et al (4), the increased risk of occupational injuries was associated with night and evening shifts. Similarly, this review suggested an exponentially increasing risk for morning and night shifts, and an increase of risk beyond the 9^th^ hour of duty (4). Recently, an association between a short time between the shifts (eg, <11 hours) and occupational injuries was found among Danish hospital workers (11, 12).

Sleepiness is regulated by the circadian and homeostatic factors and is considered to create an increased need for sleep or trouble staying awake (8, 13). Sleepiness is considered a mechanism underlying the increased risk of occupational injuries in shift work (13). As shown in many earlier studies, night shifts, early morning shifts, and a short time between consecutive shifts are associated with insufficient time to meet habitual need for daily sleep, as well as an increased sleepiness due to the requirement to stay awake at a physiologically incorrect time of the day (14–16). Shift work-related partial sleep deprivation may also accumulate over the days (17), causing impairments in alertness and cognitive performance (18).

There are several limitations in some of the earlier research examining the association of working hours with occupational injuries. In the systematic review by Fischer (4), 22 of the 24 studies on shift work and occupational injuries had cross-sectional designs that are sensitive to selection bias and residual confounding due to potential unadjusted confounders. Working during the night is associated with lower socioeconomic status, a higher physical work load, and obesity (19), all of which are associated risk factors and potential confounders for studies on occupational injuries. Several earlier studies on working hours and injuries have also been based on self-reported exposure, making them sensitive to information bias. Only few studies have utilized objective exposure data of working hours (8) and/or objective outcomes based on compensation claims (6, 20). Finally, most earlier studies on shift work and occupational injuries are based on industrial cohorts while high quality studies among nurses are rare (7).

Occupational injuries are common in hospital wards with a significance for both employee and patient safety (9, 21).

## Methods

In order to provide additional evidence and fill several methodological gaps, we used a case-crossover design with a matched-pair interval analysis to study the associations of payroll based objective working hour characteristics with the risk of a registry-based occupational injuries among hospital employees.

**Figure 1:**
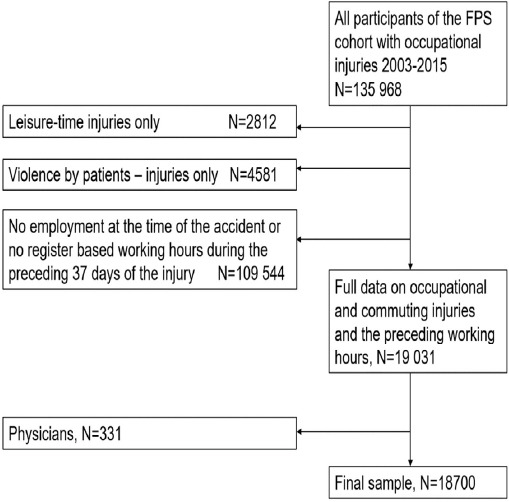
Participant selection flowchart. [FPS=Finnish public sector study.]

Based on a unique personal identification number, we linked occupational injuries of the employees of the Finnish Public Sector (FPS) cohort study from the years 2000–2015 (N=18 700, employees with ≥1 injury) with the preceding objective payroll data for 37 days before the injury (figure 1). The FPS study is one of the largest on-going open cohort studies comprising all employees of ten Finnish towns (10-town study) and six hospital districts (Work and Health in Finnish Hospital Personnel study) (22). We excluded employees who had leisure-time injuries only, ie, no occupational injuries. Leisure-time injuries are not compensated by employers and are thus not systematically found in the database of the Finnish Federation of Accidents Insurance Institutions. We also excluded employees with injuries due to violence by patients (7.9%). Although these injuries are work-related, they are often due to patient behavior (ie, alcohol-related violence during night-time) and are not likely to be associated with employees, human factors or fatigue. Physicians (N=331) were excluded due to missing information on on-call work. Of the final sample, 91% (N=18 700) were women and 9% men, the mean age of the sample was 43 (standard deviation 16–71) years. The Ethics Committee of the Hospital District of Helsinki and Uusimaa approved the study (HUS 1210/2016).

### Working hour data

The payroll-based working hour data were retrieved from a shift scheduling program Titania® used for shift scheduling and payroll in the municipal health and social sector in Finland (23). The employee data included personal identification number with information on age and sex, and the daily starting and ending times of the work shifts, including both the draft shift schedules and the final schedules used for payroll. The data included information on individual work and shift rota unit. The characteristics of the payroll based working hours are described in table 1, as reported also earlier (23, 24). Of the sample, 97% had morning, 66% had evening, and 23% had night shifts. Shift rotations with evening and night shifts were irregular, the order of the work shifts and free days varying from week to week. The shift rota tables of the employees were established for each three-week period in all hospital units separately, depending on the need for 24-hour services and patient work for each period. We classified the varying length and timing of the working hours into three shift categories: (i) morning (starting >03:00–<21:00 hours); (ii) evening (≥3 hours ≥18:00–<02:00 and not categorized as a night shift); and (iii) night (≥3 hours 23:00–06:00 hours) as described earlier (24). We also created categories for long work shifts (≥12 hours), quick returns (<11 hours between two shifts) and long weekly working hours (>40, >48 or >55 hours/week) (23, 24).

**Table 1:** Characteristics of the working hours during the control time window of 30 days (N=18 700).

	No of subjects	%	Cumulative %
Morning shifts			
	587	3.1	3.1
1–5	2981	15.8	19.1
6–10	4820	25.8	44.9
11–19	7717	41.3	86.1
20–26	2595	13.9	100
Evening shifts			
0	6282	33.6	33.6
1–5	6524	34.9	68.5
6–10	5009	26.8	95.3
11–23	885	4.7	100
Night shifts			
0	14 385	76.9	76.9
1–5	3359	18.0	94.9
6–10	676	3.6	98.5
11–20	1280	6.8	100
s≥12 hour shifts			
0	16 153	86.4	86.4
1–5	2521	3.6	99.3
6–10	92	0.5	99.8
11–19	35	9.2	100
Quick returns (<11 hours)			
0	9924	53.1	53.1
1–5	8143	43.5	96.6
6–10	630	3.4	99.9
11–14	3	0.0	100
≥40 hour weeks			
0	12 277	65.7	65.7
1	4636	24.8	90.4
2	1502	8.0	98.5
3	263	1.4	99.9
4	22	0.1	100
≥48 hour weeks			
0	17 431	93.2	93.2
1	1144	6.1	99.3
2	114	0.6	99.9
3	9	0.1	100
4	2	0.0	100
≥5 consecutive night shifts			
0	17 431	98.2	98.2
1	224	1.2	99.4
2	106	0.6	99.9
3	16	0.1	100

### Occupational injury data

Occupational injury data was obtained from the Finnish Federation of Accidents Insurance Institutions. In Finland, any costs (health costs, sickness absence, permanent damage) of occupational work-site and commuting injuries are compensated via a statutory insurance system; but injuries without any monetary costs are also required by law to be reported to the employer, who then passes on the information via the insurance companies to the Finnish Federation of Accident Insurance Institution. The injury data includes information on the date of the injury, categorization (work/commuting/leisure) and the reported external cause, location and type of the injuries (eg, falling, crashes, violence, physical load). The included leisure-time injuries are, however, only a small part of all leisure-time injuries in Finland, while occupational injuries are systematically included. Among the occupational injuries, 71% of the injuries take place during the working hours and 29% during commuting. Injuries due to sudden physical strain (29%), falling or slipping (24%), and sharp objects like needles (17%) are the most frequent.

### Statistical methods

A case-crossover design was used to assess the association between a work-related injury and various characteristics of work shifts using a matched-pair interval approach. The case-crossover approach has been shown to be an efficient design to examine transient risk factors related to injury causation (25, 26). This within-subject design has the advantage of controlling the known and unknown confounders that are different between the subjects but do not vary over time. The case-crossover approach has been previously used to study the effects of sleep and working hours on traffic accidents (27).The effects of payroll-based irregular work hour characteristics on accidental injuries (12) and sickness absence (28) have been studied before using the case-crossover design.

The basic requirements for the case-crossover design in injury research are related to the requirements of the injuries being acute with a known onset time, the exposures being transient and the possibility of the injured cases to serve as their own control (29, 30). The hazard and control windows need to be selected a priori, and the control window needs to show variability (ie, transience) in the exposure. In this study, the requirements for the known exposure and outcome timing were fulfilled based on the register data of working hours (exposure) and the information on the timing of the occupational injuries (outcome) in relation to the work shifts of all days. The study question also satisfies the requirement that the exposures be intermittent and exhibit transient effects (ie, the case moves across periods of varying exposure), as there was high transience in the shift patterns of the workers. The shift rotation tables of employees were planned at health and social sector units for three-week periods in each unit separately, hence the number of day, evening and night shifts as well as their order thus varying not only within the three week periods but also between the weeks.

In the matched-pair interval analysis, a pair of hazard and control intervals contributed by the same subject are compared. The injury risk was evaluated for three separate hazard windows: for the day of the injury, the preceding day of the injury day, and the seven preceding days of the injury day. Our hypothesis for the possible pathway from exposure of the working hours to the injury is based on increases in sleepiness during work shifts that interfere with the circadian and/or homeostatic processes, such as morning and night shifts (31). Based on this, our first hazard window was taken from the same day on which the first injury was reported. A work shift may cause sleepiness during the following day, too, especially when there is insufficient opportunity to recover through sleep between the shift and the following day, such as in connection with night shifts and quick returns (17, 32, 33). For that reason, the second hazard window was selected one day after the exposure. Finally, it is possible that night shifts or other nonstandard work hour arrangements cause accumulation of sleepiness and impairments in cognitive performance, thus causing a progressive injury risk over several days (4, 18). To study this, the last hazard window included seven preceding days of the injury. We did not use longer periods, eg, one month, as the characteristics of the current shift system were balanced every three weeks. This means hazard windows of several weeks would have decreasing variability compared to one-week periods that differed more in relation to the length, timing and intensity of the working hours.

The hazard period for the date of the injury was defined as the calendar day including the morning or evening shift (00:00–24:00) or the end of the night shift (from 00:00–24:00, the night shifts starting mostly between 20:00–23:00 during the preceding date and ending at around 07:00 hours the following date). For night shifts, the hazard window following the night shift was the date after the end of the night shift. If the injury took place during a specific shift, it was coded as 1 (“yes”), otherwise the date was coded as 0 (“no”). The control window was always the same weekday including any work shift occurring seven dates earlier than the hazard window. If the control window one week earlier did not include any work shift, a new control window was looked for from the earlier week on the same weekday, up to the maximum of four weeks apart from the injury. If no control window was found during the preceding four weeks, the observation was assigned as missing.

For the hazard window of the preceding seven dates of the date of the injury, the control window consisted of the previous seven dates of the hazard window, starting from the same weekday as the hazard window. If the control window one week earlier did not include any work shifts, the control window was taken from the earlier week, etc. up to the maximum of four weeks before the injury. The first incidence of an occupational injury alone for each subject was considered. We estimated odds-ratios (OR) and 95% confidence intervals (CI) for the risk of injury in the hazard windows compared to the control window using conditional logistic regression analysis.

## Results

Table 2 shows that there was a statistically significantly, elevated risk for an occupational injury during the morning (OR 1.06, 95% CI 1.01–1.11) and evening (OR 1.09, 95% CI 1.03–1.14) shift, but not during the night shift (OR 0.98, 95% CI 0.88–1.08). Using the preceding day as the hazard period, injury risk was not increased following a morning or an evening shift but was increased during a work shift following a night shift (OR 1.33, 95% CI 1.17–1.52). Specific shift combinations within the injury hazard windows showed for the most part non-significant associations with injuries, except for a decreased risk for the combination of a morning shift (during the preceding day of the start of the night shifts) and a night shift (OR 0.63, 95% CI 0.43–0.93), and a decreased risk for the combination of an evening and night shift (OR 0.69, 95% CI 0.48–0.99). Long work shifts with ≥12 hours were associated with an increased risk during the shift (OR 1.23, 95% CI 1.06–1.42).

**Table 2:** Matched-pair interval analysis of morning (M), evening (E) and night (N) shifts, long work shifts, and shift combinations with the risk of occupational injury ^a^. [OR=odds ratio; CI=confidence interval.]

	Injury day				Preceding day				The combination of the preceding and injury day			
		
	Hazard window (N)	Control window (N)	OR	95% CI	Hazard window (N)	Control window (N)	OR	95% CI	Hazard window (N)	Control window (N)	OR	95% CI
Morning	12 566	12 432	1.06	1.01–1.11	8851	9074	0.98	0.92–1.04				
MM									7289	7260	1.01	0.96–1.07
EM									1890	1812	1.05	0.98–1.12
NM									10	8	1.25	0.44–3.69
Evening	4293	4004	1.09	1.03–1.14	3258	3107	1.01	0.95–1.07				
ME									1145	1080	1.07	0.98–1.16
EE									1283	1218	1.06	0.98–1.16
NE									14	8	1.68	0.66–4.64
Night	798	806	0.98	0.88–1.08	673	503	1.33	1.17–1.52				
MN									46	72	0.63	0.43–0.93
EN									53	77	0.69	0.48–0.99
NN									220	252	0.86	0.71–1.04
≥12 hours	440	364	1.23	1.06–1.42								

a Injury day, the preceding day, and the combination of the preceding and injury day during the hazard window compared with a control window 1-4 weeks earlier without the injury.

Work-related injury risk was also estimated based on the number of morning, evening or night shifts during the preceding seven days (table 3). Having only 1 morning (OR 0.94, 95% CI 0.89–0.99) or 0 evening (OR 0.93, 95% CI 0.89–0.98) shifts during the seven days ending in an occupational injury was associated with a significant decrease in injury risk. However, the risk increased significantly following a week including ≥5 morning shifts (OR 1.12, 95% CI 1.07–1.18), or following a week including ≥3 evening shifts (OR 1.15–1.22) preceding the injury. For night shifts, the risk was increased in the case of having only 1 night shift (OR 1.12, CI 1.01–1.23) during the preceding week, but became lower and insignificant according to a higher number of night shifts during the preceding week (table 3).

**Table 3:** Matched-pair interval analysis of the number ^a^ of morning (M), evening (E) and night (N) shifts during the preceding 7 days with occupational injuries. [OR=odds ratio; CI=confidence interval.]

M/E/N shifts (N)	Morning shifts				Evening shifts				Night shifts			
		
	Hazard window (N)	Control window (N)	OR	95% CI	N	N	OR	95% CI	N	N	OR	95% CI
0	1901	1985	0.94	0.88–1.00	10 354	10 683	0.93	0.89–0.97	18 837	18 774	1.03	0.97–1.10
1	2881	3032	0.94	0.89–0.99	4398	4423	0.99	0.95–1.04	912	830	1.12	1.01–1.23
2	3943	3923	1.01	0.96–1.06	3742	3720	1.01	0.96–1.06	874	920	0.94	0.85–1.03
3	3876	3864	1.00	0.96–1.06	1765	1557	1.15	1.07–1.23	350	409	0.85	0.74–0.98
4	3342	3468	0.96	0.91–1.01	688	618	1.12	1.00–1.26	183	209	0.87	0.70–1.08
≥ 5	4804	4418	1.12	1.07–1.18	321	266	1.22	1.03–1.44	111	125	0.81	0.59–1.12

a The number of work shifts during the hazard window of 7 days before the injury day compared with a control window of 7 days 1–4 weeks earlier.

The number of quick returns during the preceding week was not significantly associated with the injury risk (OR 1.40, 95% CI 0.68–2.89 for two quick returns during the preceding week, table 4). Similarly, the length of the total weekly working hours of the preceding week was not associated with the risk for injury for the working weeks of >40 hours (OR 0.99, 95% CI 0.94–1.04, N=4570), for the working weeks of >48 hours (OR 1.00, 95% CI 0.92–1.08, N=1540), or for the working weeks of >55 hours (OR 1.01, 95% CI 0.91–1.13, N=926).

**Table 4:** Matched-pair interval analysis of the number a of quick returns (<11 hours between the shifts) with occupational injuries during the preceding 7 days. [OR=odds ratio; CI=confidence interval.]

No of quick returns	Hazard window (N)	Control window (N)	OR	95% CI
0	18 636	18 636	0.97	0.86–1.09
1	36	42	0.84	0.53–1.35
2	18	13	1.40	0.68–2.89
3	10	9	1.13	0.45–2.76
≥4	10	10	1.00	0.41–2.46

^a^ The number of quick returns during the hazard window of 7 days compared with a control window of 7 days 1–4 weeks earlier.

Since the etiology of work time and commuting injuries may be different, we conducted a sensitivity analysis on the association of different work shifts with occupational injuries excluding the smaller group of commuting injuries. The main results were similar to the combined data: the risk for occupational injuries was elevated during the evening shifts (OR 1.15, 95% CI 1.09–1.23, N=3173) and following the night shifts (OR 1.44, 95% CI 1.24–1.69, N=623). The risk for injuries was not statistically significant during the night shifts (OR 1.06, 95% CI 0.94–1.19, N=623) or during the morning shifts (OR 1.01, 95% CI 0.95–1.07, N=8159).

## Discussion

This study of over 18 000 hospital employees with occupational injuries linked with comprehensive objective working hour data utilized a case-crossover design with a matched-pair interval analysis to investigate the associations of working hour characteristics with occupational injuries. The results indicate an increased injury risk following ≥5 morning shifts and following ≥3 evening shifts during the preceding week. For the night shift, the injury risk increased only during the following work shift but not according to the number of night shifts during the preceding week. The risk for occupational injuries increased during long work shifts.

We observed an association between the number of evening shifts and occupational injuries, with an increasing pattern in relation to the number of preceding evening shifts and the risk for injury. Since the observed risks for injuries between the morning, evening and nights shifts – as well as in between the different number of preceding work shifts – are not directly comparable, the results need to be interpreted with caution. However, the findings of the association of evening shifts and injuries does not seem to be in line with the meta-analysis of Fischer et al (4) but is consistent with the recent Danish payroll study (8, 12). The Danish study also found an increasing trend for the association between the number of evening shifts during the preceding week and the risk for injury. For the night shift, we found an increased risk for occupational injuries only during the days following the night shift but not during the night shift days. The Danish payroll study (8) found a similar association between the number of night shifts and injury risk, but this study did not differentiate injury risk for the periods during or following the night shifts. The Danish study focused also on the more serious injuries, based on database requiring contacts to emergency rooms, and studied the risk in relation to any injuries, whether taking place during working hours, commuting or leisure-time. The lack of information on leisure-time injuries in our study may have weakened the observed association between night shifts and the risk for occupational injuries. Injuries were more frequent following the night shifts, suggesting that they might take place also during free-time reserved for recovery.

We found that occupational injuries were most frequent following the weeks of only one night shift, and least frequent following the weeks with three night shifts. These findings are not consistent with earlier studies, most of them in industry (4). Occupational injury risk may be the highest during the first night shift due to higher odds for severe sleepiness, as found in several studies (14, 15, 34). On the other hand, insufficient sleep during the following night shifts may cause cumulative fatigue that can increase the risk for injury. We found that only night shifts, not the morning or evening shifts, were associated with occupational injuries following the shifts. This, however, may be due to the observed shorter sleep length after the night shifts, compared to average sleep length after the morning and evening shifts (16, 17).

The finding indicating occupational injuries were the most frequent following the week including 3 night shifts, but not following the week of ≥5 night shifts, cannot be explained by a lower cumulative sleep deprivation. The used case-crossover design compares exposure within individuals with the benefit of excluding bias due to the possible inter-individual differences. This means that the observed null findings in relation to the possible increased risk due to several night shifts are not biased due to individual differences. However, the lower observed risk among those having several night shifts may be due to having a smaller self-selected group of, for the most part, permanent night workers. The group having occupational injuries following ≥5 night shifts was smaller (N=111) than the group having the maximum of 3 (N=350) or only 1 (N= 912) night shift during the preceding week of the injury. The OR stratified according to the number of work shifts are thus not comparable to each other, particularly in a situation in which an employee performs many night shifts. Furthermore, we should not exclude the possibility that an increase in the number of consecutive night shifts in a non-selected group of irregularly rotating night shifts might cause an increase in the risk for occupational injuries (35, 36).

The reason for the increased risk for injuries during the evening shifts in hospital work is unclear. It is well known that sleepiness and fatigue in hospital work is lower during the evening than the night shifts, as shown also by us in the same population using a field study (16, 37). The evening shifts usually started at 13:00–13.59 while the night shifts most frequently started at 20.00–20.59, being up to three hours longer than the evening shifts (24). Based on our earlier joined study, morning shifts accounted for 8% of all operational hours (by each hour of the shift), while evening hours made up 3% and night shifts only 1% (24). The need for workforce is thus much more limited in the evenings and especially during the nights, reflecting probably variation in work demands and the availability of the work force. The need for patient care and possibly also for safety-sensitive operations is the lowest during the nights, when most patients in hospital wards sleep. During the evenings, staffing level decreases remarkably from daytime, but it is possible that the work demands for each employee are high due to patients being awake and needing hospital care. We also found a small increased risk for the morning shifts that was not significant in the sensitivity analysis after excluding commuting injuries. This suggests that commuting, possibly due to travelling during the rush hours or eg, more slippery pavements in the early morning together with perceived sleepiness, could be the reason for the observed risk. The observed decreased risk for occupational injuries for the shift combination of a morning shift during the preceding day, and a night shift during the following day is supported by some earlier observations for a lengthened sleep, a high percentage of napping and a low number of dozing off in a morning-night shift combination (38).

We found that long shifts were associated with occupational injuries. On the other hand, the number of quick returns during the preceding week was not associated with the risk for injury. The findings that long work shifts are linked with occupational injuries is mostly supported by the earlier studies, reporting on the association of long working hours with decreased safety and lower quality of care (4, 21, 39). A short lapse of time between the shifts, normally the criteria being <11 hours according to the EU Working Time Directive, has been found earlier to be related to an increased risk for occupational injuries among hospital employees (12, 40, 41). For example, the Danish payroll data among hospital workers found a 39% increased risk for occupational injuries in quick returns compared to shift intervals of 15–17 hours, adjusted to year, season, age, sex and occupation. The risk decreased to 17% in the fully adjusted model (11). Quick returns with the length 6–8 hours were not associated with an increased risk in the full statistical model, the risk consisting mostly from quick returns of only 1–5 hours (split shifts). This would mean our results are not fully in disagreement with the Danish payroll study. However, we could not find significant associations between quick returns and the number of injuries. This may be due to the small number of quick returns in this sample, and possible interactions of quick returns with some other characteristics of working hours. For example, the percentage of short shift intervals correlated negatively (correlation coefficient 0.41) with long spells of consecutive night shifts in our earlier study (23).

The strengths of this study are the use of objective registry data for both daily working hours and occupational injuries and the benefit of having a large and representative sample of hospital employees without a loss of follow-up. Our results add to some earlier registry studies with the design of the study and the analysis of several working hour characteristics in three separate hazard windows in order to get a comprehensive overview of the detailed working hour characteristics relevant to occupational safety. The use of payroll data offers the advantage of having access to precise information without recall bias or attrition (23). The use of payroll data is also optimal for the analysis of irregular shift systems with transient working hours. The case-crossover approach used is highly appropriate when examining transient risk factors related to injury causation (25). Since the employees are compared to themselves, the design controls for all measured or unmeasured confounders that do not vary between the hazard and control windows. The design thus automatically controls for between-employee confounders, work-related differences, and time of the year.

Overall, our study and the use of the case-crossover study design has some limitations (25). We could not control for the potential shift-dependent variation in work demands and tasks associated with the risk for injuries. The differences in occupational injury risk between the morning, evening and night shift days may be due to differences in work tasks. The exact time of the day of the injury was not known, but having the minute-based follow-up of the true working hours, we could locate the timing of the injuries to different times of the day. The OR for morning, evening and night shifts are not directly comparable due to the higher number of subjects, and possible self-selection. Since night shifts were less frequent than morning and evening shifts, as well as the shift combinations associated with night shifts, the probability of finding a hazard window from the preceding week was lower, decreasing the statistical power. This also increases the probability for confounding due to the possibility of individual differences changing over time (eg, a respiratory infection) between the control and hazard windows. However, we could decrease the bias by limiting the lag to a maximum of four weeks.

We had to exclude the leisure-time injuries due to the data not being representative of the study population. Since leisure-time injuries can also partly depend on preceding work shifts in addition to the occupational injuries (defined as injuries during working hours or commuting, according to the Finnish legislation). This is likely to attenuate the observed association of night shifts and the risk for injuries since we found a significantly increased risk especially after, but not during the night shifts. As for commuting injuries, albeit there is control of between-person confounders in this study design, one limitation is the control of within-person confounding which is still possible for multiple, correlated transient factors that change over time within a subject. For example, if a driver, feeling tired, uses concurrently a mobile phone (ie, distraction) while moving through hazardous road conditions, this confounding would be uncontrolled and could still be a threat to the internal validity (29). Finally, since the sample is based on hospital employees only, the results should not be directly generalized to other sectors. Future studies should use even large samples in order to stratify the results according to the different types and characteristics of the injuries.

Several practical conclusions can be made based on the obtained results. Preventive safety measures on irregular shift work arrangements should pay extra attention on evening shifts, which were shown to be the highest risk period for injuries. Long work shifts, long spells of consecutive evening shifts and single night shifts, which were shown to have an increased risk for occupational injuries, should be avoided or minimized if possible. Having a morning or evening shift during the preceding day of the starting time of the first night shift might offer possibly better recovery before the becoming spells of nights, as previously reported (38).
